# Visual evoked potential importance in the complex mechanism of amblyopia

**DOI:** 10.1007/s10792-013-9734-6

**Published:** 2013-02-16

**Authors:** Regina Halfeld Furtado de Mendonça, Stefania Abbruzzese, Bruna Bagolini, Italo Nofroni, Eliana Lucia Ferreira, James Vernon Odom

**Affiliations:** 1 Department of Ophthalmology, “La Sapienza” University, Rome, Italy; 2 Grupo de Pesquisa em Inclusão, Movimento e Ensino à Distância (Center of Distance Education), Universidade Federal de Juiz de Fora (UFJF), Campus Universitário S/N, Bairro Martelos, Juiz de Fora, MG CEP 36036-900 Brazil; 3 Department of Statistic, “La Sapienza” University, Rome, Italy; 4 Department of Ophthalmology, West Virginia University Eye Institute, Morgantown, WV USA

**Keywords:** Amblyopia, Anisometropia, Strabismus, Visual evoked potential (VEP)

## Abstract

To compare the visual evoked potential (VEP) responses of amblyopic eyes with VEP responses of sound eyes in amblyopic children. A study of 65 amblyopic children with pattern-reversal VEPs elicited by checkerboard stimuli with large, medium and small checks. The children were classified into three groups: Group A, 22 children with anisometropic amblyopia; Group B, 16 children with exotropic strabismic amblyopia; and Group C, 27 children with esotropic strabismic amblyopia. Visual acuity (VA) was significantly worse in the amblyopic eye as compared to the sound eye. However, no statistically significant difference was found between the amblyopic and sound eye of amblyopic children in the three groups for VEP P1 amplitude and latencies for any check sizes. VEP is a very important tool in understanding the complex amblyopic mechanism. Although the sound eye has superior VA, the absence of differences in VEP P1 amplitudes and latencies demonstrate the functional abnormality of the eye considered ‘good’. More studies are necessary to explain why the sound eye in amblyopic children cannot be considered completely normal. Special attention should therefore be paid to amblyopic treatment, as patching can have a negative effect on the sound eye.

## Introduction

Special attention must be given to amblyopic patients as they are exposed to the potential risk of becoming blind, generally by trauma of the healthy eye at a markedly higher rate as compared with the general population [1]. The primary causes of functional amblyopia are a difference between the refractive power of the two eyes, termed anisometropia, and a misalignment of the visual axis of one eye compared to that of the other, termed strabismus [2]. Amblyopia can be unilateral as manifest strabismus (esotropia, exotropia and hypertopia), anisometropia (anisohypermetropia, anisomyopia, anisoastigmatism, aniseikonia) and visual deprivation (cataract, complete ptosis, opaque cornea, hyphema, vitreous clouding, prolonged uncontrolled patching, prolonged unilateral blepharospasm and prolonged unilateral atropinization) or bilateral caused generally by visual deprivation (cataracts of equal density, high uncorrected hypermetropia and motor type nystagmus) [3]. Two mechanisms for amblyopia have been suggested. The first is lack of adequate visual stimulation during infancy, causing visual deprivation and the second is based on abnormal binocular interaction [3]. The visual evoked potential (VEP) is a method whereby changes of the electrical potentials generated by the brain and recorded over the occipital cortex as a consequence of visual stimulations, are studied. This is mostly used in lesions of optic nerve and visual pathways. The VEP is an efficient, objective and practical diagnostic procedure which monitors the activity of the visual system at the level of the occipital visual cortex. It originates from the massed activity of a large number of cortical neurons [4].

The use of VEP in amblyopia has been reported previously [5]. The relationship between VEP and visual acuity (VA) has been the subject of many articles [6, 7]. Comparison between Teller Acuity Cards (TAC) and VEP demonstrate that TAC testing gives poorer acuity scores than VEP testing in children with moderate to severe developmental delay [8]. Others pointed out that a clear correlation between VA and VEP amplitude may not be present in amblyopia, since this condition could be the result of several types of disturbance at different levels in the visual system [9]. Amblyopia is not a static condition but has a strong dynamic component since its severity can be modified by the type of stimulation received by the sound eye [3]. Consequently, the responsibility of the ophthalmologist does not end by suggesting occlusion, but requires following the treatment intensively [10]. We decided to give special attention to amblyopic children using VEP to study and monitor those cases.

## Subjects and methods

Sixty-five amblyopic children from 2 to 13 years underwent comprehensive, complete ophthalmologic examinations, including VEPs, in the ambulatory Electrophysiology Suite of the Ophthalmic Clinic of the Faculty of Medicine of the ‘Università Degli Studi La Sapienza’ in Rome. All patients had been previously examined by the Orthoptic Department. All the parents had provided written consent and confirmed that the child was not previously received any amblyopic treatment. As orthoptic and electrophysiology tests were routine examinations, it was not necessary to obtain approval by the ethics board in the hospital.

The patients were classified into three groups Group A, 22 children (33.85 %) with anisometropic amblyopia; Group B, 16 children (24.61 %) with exotropic strabismic amblyopia; and Group C, 27 children (41.54 %) with esotropic strabismic amblyopia. The patients were tested before amblyopia therapy.

Best-corrected monocular visual acuity was measured by covering one eye at a time and measuring the acuity of the uncovered eye. If the child could read, a back-illuminated Snellen chart with decimal notation X 10 (Sbisà) placed 6 m from the child was used to measure visual acuity. If the child was small and was unable to read but could interpret symbols, a back-illuminated tumbling ‘E’ (one optotype shown in four different positions) was used.

The correspondences between VA from decimal notation and 20/20 notation are 0.1 (20/200); 0.2 (20/100); 0.4 (20/50); 0.5 (20/40); 0.6 (20/30); 0.8 (20/25) and 1.0 (20/20).

The diagnosis of amblyopia was based on VA measurement. In the strabismic amblyopia associated with exotropia and esotropia we had a manifest deviation of one eye (labeled as amblyopic eye) and a fixation of the other eye (labeled as sound eye). Microstrabismus was present in all children with anisometropic amblyopia that were evaluated by the four-prism diopter base-out test. The worse seeing eye was labeled as the amblyopic eye and the better seeing eye was labeled as the sound eye.

Pattern-reversal VEPs were recorded using techniques based on those previously described using the ‘Biomedica Mangoni’ system [11]. Patients were seated 1 m from a 17 in. monitor (19.5°). Mean luminance of the display was 80 cd/m^2^. Contrast between black and white squares was 90 %. A reversal rate of two reversals per second was used. The patients underwent a clinical protocol with three check sizes large checks sizes (120 min of arc); medium checks sizes (42 min of arc) and small checks sizes (12 arc min).

The electroencephalogram was amplified 50,000 times with a ½ amplitude bandpass of 1–50 Hz with a 50 Hz notch filter in place. VEPs were the average of 100 epochs of the electroencephalogram recorded monocularly from each eye separately (amblyopic and sound eye) for each check size. Fixation was monitored by an observer and data collected only when the child was looking at the pattern.

A pattern-reversal-elicited VEP waveform comprises a negative peak N75, which occurs at about 75 ms, followed by a positive peak P100 which occurs at about 100 ms. The VEPs were analyzed considering the P100 latency (measured from 0 ms to the highest point of the peak) and the P100 amplitude (measured from N75 to P100).

Statistical analysis was performed using a commercially available statistical software package (SPSS for windows, version 10, SPSS, Chicago, IL, USA). Analyses were conducted separately for the amblyopic and the nonamblyopic (or sound) eyes. *T*-tests and paired samples tests were employed to compare VA and P100 from the different checks sizes (amplitudes and latency) of amblyopic eyes with sound eyes in each group separately. A *p* value of ≤0.05 was considered statistically significant.

## Results

The mean and standard deviation of age (in months) of Group A was 84.77 ± 24.87, of Group B was 114.69 ± 30.80 and for Group C was 80.22 ± 19.99. The mean ages differed statistically among the groups (*p* = 0.001).

The paired samples test, comparing amblyopic eyes with sound eyes of all patients together from Groups A, B and C shows a statistically significant difference (SSD) concerning mean VA between the amblyopic and sound eye of amblyopic children in all groups (Table 1).

**Table 1:** Comparison of mean visual acuity of the amblyopic eye with mean visual acuity of the sound eye in the three groups

Groups	A	B	C
Visual acuity			
Mean amblyopic eye	0.695	0.700	0.548
Mean sound eye	0.977	0.888	0.944
SD of the difference	0.232	0.150	0.270
	*p* = 0.0001*	*p* = 0.0001*	*p* = 0.0001*

Mean of visual acuity in decimal notation

*Group A*, children with anisometropic amblyopia; *Group B*, children with exotropic strabismic amblyopia; *Group C,* children with esotropic strabismic amblyopia

* A *p* value of ≤0.05 was considered statistically significant

Taking large (120′), medium (42′) and small (12′) checks into consideration, no SSD in P1 amplitude and latency was detected between the amblyopic and sound eye of amblyopic children in all three groups (Tables 2, 3, 4). No SSD concerning P1 amplitude and latency for any stimuli was detected between the amblyopic and sound eye of amblyopic children in all the three groups.

**Table 2:** Comparison of the mean P1 amplitude and latency with 120 arc min of the amblyopic eye with the mean P1 amplitude and latency of the sound eye in the three groups

Groups	A	B	C
Amplitude 120 arc min (μV)			
Mean amblyopic eye	21.96	14.13	15.17
Mean sound eye	19.90	13.8	15.99
SD of the difference	6.10	5.26	5.16
	*p* = 0.128	*p* = 0.881	*p* = 0.075
Latency 120 arc min (ms)			
Mean amblyopic eye	106.45	110.20	106.19
Mean sound eye	106.14	107.19	105.36
SD of the difference	6.29	6.59	4.18
	*p* = 0.815	*p* = 0.122	*p* = 0.677

*Group A,* children with anisometropic amblyopia; *Group B*, children with exotropic strabismic amblyopia; *Group C*, children with esotropic strabismic amblyopia; *μV,* microvolts; *ms,* milliseconds

**Table 3:** Comparison of the mean P1 amplitude and latency with 42 arc min of the amblyopic eye with the mean P1 amplitude and latency of the sound eye in the three groups

Groups	A	B	C
Amplitude 42 arc min (μV)			
Mean amblyopic eye	18.89	11.87	11.53
Mean sound eye	17.47	12.08	12.87
SD of the difference	8.01	5.11	5.27
	*p* = 0.414	*p* = 0.870	*p* = 0.054
Latency 42 arc min (ms)			
Mean amblyopic eye	114.36	117.13	117.59
Mean sound eye	113.55	115.13	155.56
SD of the difference	7.93	14.23	8.08
	*p* = 0.633	*p* = 0.582	*p* = 0.365

*Group A,* children with anisometropic amblyopia; *Group B*, children with exotropic strabismic amblyopia; *Group C*, children with esotropic strabismic amblyopia; *μV,* microvolts; *ms,* milliseconds

**Table 4:** Comparison of the mean P1 amplitude and latency with 12 arc min of the amblyopic eye with the mean P1 amplitude and latency of the sound eye in the three groups

Groups	A	B	C
Amplitude 12 arc min (μV)			
Mean amblyopic eye	11.63	7.5	9.32
Mean sound eye	12.02	7.99	9.90
SD of the difference	6.26	2.82	6.04
	*p* = 0.772	*p* = 0.500	*p* = 0.377
Latency 12 arc min (ms)			
Mean amblyopic eye	118.68	121.63	114.00
Mean sound eye	118.27	116.31	113.96
SD of the difference	6.75	10.48	25.95
	*p* = 0.779	*p* = 0.061	*p* = 0.863

*Group A,* children with anisometropic amblyopia; *Group B*, children with exotropic strabismic amblyopia; *Group C*, children with esotropic strabismic amblyopia; *μV,* microvolts; *ms,* milliseconds

## Discussion

Although the light flash VEP is generally a highly reliable method for many different diagnostics [12], in the present study, only the pattern VEP was used because the changes connected with amblyopia are most easily demonstrated with pattern stimulus [9] and the magnocellular layers of the lateral geniculate nucleus may be relatively spared in amblyopia [13].

It was expected that VEP responses of the amblyopic eye would have lower amplitude than stimulation of the normal eye [5, 14]. In the present study no SSD concerning P1 amplitude and latency for any stimuli was detected between the amblyopic and sound eye of amblyopic children in all the three groups. In amblyopic eyes, reportedly the amplitude of pattern-reversal VEPs was significantly smaller and the latency significantly longer and the magnitude of those changes correlated with the reduction of VA [13]. However, in another study, [15] only four of 10 patients (aged 10–22 years) presented with an asymmetry of >27 % between amblyopic and normal eyes. Furthermore, although a large asymmetry in amplitude was found in those cases, it was not always the normal eye that gave the greatest response. The authors considered the response of the amblyopic eye as ‘supernormal’ [15]. We suspect that differences in VEP amplitude between the amblyopic and sound eye can be related with check sizes. Large checks cannot be used to detect or monitor occlusion therapy in strabismic amblyopes since, before treatment, most of them showed little or no interocular difference in the P1 amplitude [16]. Interocular VEP and reaction time findings in anisometropes are similar to those of normal subjects while those of strabismic show an increased interocular reaction time delay, suggesting that the neural insult leading to strabismic amblyopia could be more severe than that causing anisometropic amblyopia [17]. Some authors have found larger interocular VEP amplitude differences in anisometropic than in strabismic amblyopes [16] while other studies have shown that VEP amplitude could not distinguish between the two types of amblyopia [17]. In the present study, the interocular VEP and reaction time in the anisometropic (Group A) and strabismic amblyopia groups (Groups B and C) demonstrated no SSD between the amblyopic eye and the sound eye.

The correlation between VA and VEP amplitude has been the subject of many articles [6, 7, 9]. In the present study, no relationship between mean VA and mean P100 amplitude was present in all groups together or separately.

As no SSDs were detected in VEP amplitude or latency for any check sizes between the amblyopic and sound eyes in any groups (Tables 2, 3, 4), these findings are consistent with the suggestion that the sound eye in amblyopic patients is not really normal.

More studies are necessary to explain why the sound eye in amblyopia cannot be considered completely normal; however, special attention should be paid to amblyopic treatment, as patching can have a negative effect on the sound eye [18].
